# Correction: Protein-protein interactions enhance the thermal resilience of SpyRing-cyclized enzymes: A molecular dynamic simulation study

**DOI:** 10.1371/journal.pone.0306009

**Published:** 2024-06-27

**Authors:** The PLOS One Staff

## Notice of republication

This article was republished on 10^th^ June 2024 to correct an author name error. The publisher apologizes for the error. Please download this article again to view the correct version.

The second author’s name was spelled incorrectly. The correct name is: Dengming Ming.

## Supporting information

S1 File Originally published, uncorrected article. (PDF)

S2 File Republished corrected article. (PDF)
