# Supplementary figures and images for: Protein-protein interactions enhance the thermal resilience of SpyRing-cyclized enzymes: A molecular dynamic simulation study
Source: PLoS One. 2022 Feb 17;17(2):e0263792. doi: 10.1371/journal.pone.0263792 (PMC8853484; doi:10.1371/journal.pone.0263792)

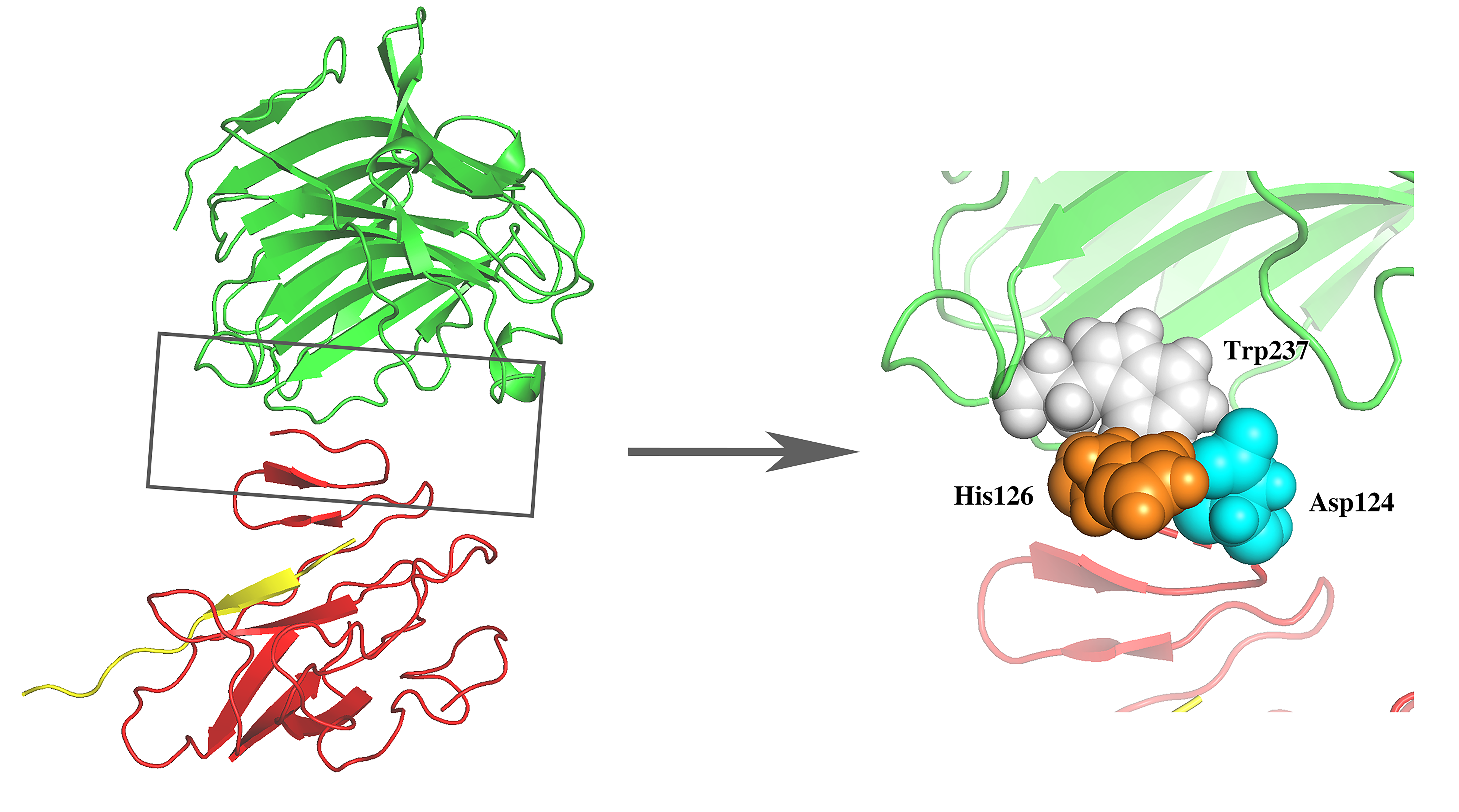

Supplement: S4 Fig — The protein structure calculated by the ZDOCK program (left), in which green is lichenase, yellow is SpyTag, red is SpyCatcher, and the protein-protein contact interface shown in the box (right). (PNG) [file pone.0263792.s004.png]
