# Supplementary material for: Protein-protein interactions enhance the thermal resilience of SpyRing-cyclized enzymes: A molecular dynamic simulation study
Source: PLoS One. 2022 Feb 17;17(2):e0263792. doi: 10.1371/journal.pone.0263792 (PMC8853484; doi:10.1371/journal.pone.0263792)
Supplement: S1 Table — The amino acid sequence of the random complex was aligned with that of the SpyRing complex, so the same amino acid has the same sequence number, that is, the sequence number of the SpyRing complex. (DOCX) [file pone.0263792.s005.docx]

**S1 Table. The difference between the Tag/Catcher-enzyme interface of the simulated SpyRing complex structure and that of the random docking complex.** The amino acid sequence of the random complex was aligned with that of the SpyRing complex, so the same amino acid has the same sequence number, that is, the sequence number of the SpyRing complex.

| Group | | Tag/catcher | lichenase | Interaction |
| --- | --- | --- | --- | --- |
| For simulation | Wild type | Asp30 | Asn288 | VDW |
|  |  | Met31 | Asn288 | VDW |
|  |  | Met31 | Asn304 | VDW |
|  |  | Thr32 | Asn288 | Hbond |
|  |  | Glu34 | Lys291 | Hbond |
|  |  | Lys42 | Asn288 | Hbond |
|  |  | Arg46 | Thr235 | VDW |
|  |  | Lys122 | Lys137 | Hbond |
|  |  | Lys122 | Lys347 | Hbond |
|  |  | Asp124 | Gly205 | Hbond |
|  |  | Asp124 | Tyr206 | Hbond/VDW |
|  |  | Asp124 | Pro310 | VDW |
|  |  | Asp124 | Lys347 | Ionic/Hbond |
|  |  | His126 | Pro231 | VDW |
|  |  | His126 | Thr309 | VDW |
|  |  | His126 | Thr322 | Hbond |
|  |  | His358 | Tyr206 | Pipistack/VDW |
|  |  | His358 | Pro287 | VDW |
|  |  | Val362 | Thr235 | VDW |
| For random docking | Wild type | Asp124 | Trp237 | VDW |
|  |  | His126 | Trp237 | VDW |
|  | T136K | Gly118 | Pro231 | VDW |
|  |  | Lys122 | Gln305 | VDW |
|  |  | His126 | Gly262 | Hbond |
|  |  | His126 | Asn263 | VDW |
|  | K122E | Lys119 | Asn259 | VDW |
|  |  | Ala120 | Ans259 | VDW |
|  | K122G | Gln111 | Gln305 | Hbond |
|  |  | Ala120 | Asn259 | VDW |
|  | D124G | Gly118 | Gly322 | Hbond |
|  |  | Lys119 | Asn162 | VDW |
|  |  | Ala120 | Thr321 | VDW |
