# Supplementary material for: Protein-protein interactions enhance the thermal resilience of SpyRing-cyclized enzymes: A molecular dynamic simulation study
Source: PLoS One. 2022 Feb 17;17(2):e0263792. doi: 10.1371/journal.pone.0263792 (PMC8853484; doi:10.1371/journal.pone.0263792)
Supplement: S2 Table — (DOCX) [file pone.0263792.s006.docx]

**S2 Table. The interface changes with increasing temperature (from 333 K to 348 K).**

|  | Tag/catcher | lichenase | Interaction |
| --- | --- | --- | --- |
| Wild type (333 K) | Asp30 | Asn288 | VDW |
|  | Met31 | Asn288 | VDW |
|  | Met31 | Asn304 | VDW |
|  | Thr32 | Asn288 | Hbond |
|  | Glu34 | Lys291 | Hbond |
|  | Lys42 | Asn288 | Hbond |
|  | Arg46 | Thr235 | VDW |
|  | Lys122 | Lys137 | Hbond |
|  | Lys122 | Lys347 | Hbond |
|  | Asp124 | Gly205 | Hbond |
|  | Asp124 | Tyr206 | Hbond/VDW |
|  | Asp124 | Pro310 | VDW |
|  | Asp124 | Lys347 | Ionic/Hbond |
|  | His126 | Pro231 | VDW |
|  | His126 | Thr309 | VDW |
|  | His126 | Thr322 | Hbond |
|  | His358 | Tyr206 | Pipistack/VDW |
|  | His358 | Pro287 | VDW |
|  | Val362 | Thr235 | VDW |
| Wild type (348 K) | Gln25 | Thr235 | VDW |
|  | Gln27 | Pro287 | Hbond |
|  | Gly29 | Gln286 | VDW |
|  | Gly29 | Asn288 | Hbond |
|  | Asp30 | Gln286 | Hbond/VDW |
|  | Arg46 | Gly234 | Hbond |
|  | Gly50 | Thr235 | Hbond |
|  | Lys51 | Thr235 | Hbond |
|  | Lys51 | Trp237 | VDW |
|  | Asp124 | Tyr206 | Hbond |
|  | Asp124 | Lys347 | Ionic/Hbond |
|  | His126 | Asp233 | Ionic/Hbond |
|  | His358 | Tyr206 | Pipistack/VDW |
|  | His358 | Asp233 | Hbond |
| K122G (333 K) | Gln113 | Ser139 | Hbond |
|  | Gly118 | Gln202 | VDW |
|  | Ala120 | Gly137 | VDW |
|  | Gly122 | Gly137 | Hbond |
|  | Gly122 | Lys347 | Hbond/VDW |
|  | Asp124 | Lys347 | Ionic/Hbond/VDW |
|  | His126 | Tyr206 | Pipistack/VDW |
|  | His126 | Tyr308 | Hbond |
| K122G (348 K) | Gly122 | Lys347 | Hbond |
