# Supplementary material for: Protein-protein interactions enhance the thermal resilience of SpyRing-cyclized enzymes: A molecular dynamic simulation study
Source: PLoS One. 2022 Feb 17;17(2):e0263792. doi: 10.1371/journal.pone.0263792 (PMC8853484; doi:10.1371/journal.pone.0263792)
Supplement: Attachment — Submitted filename: Replies to review comments_lichenase.docx [file pone.0263792.s007.docx]

Reviewer #1: This article applied MD simulations to study the protein-protein interactions and is aimed to demonstrate these interactions can enhance the thermal resilience of SpyRing-cyclized enzymes. They mutated some residues to study their role in the protein-protein interactions. However, I wouldn’t like to this paper to be published due to the following reasons:

1. Abstract is too long. The authors should remove some redundant research background and summarize the most important information of this work in abstract.

We had shortened the abstract as suggested.

2. The arrangement and English writing of this manuscript should be greatly improved for better understand of the work.

We spent a lot of energy to shape the writing and believed that it has become better.

3. Some typos in the method part should be corrected. For example, “Long range electrostatics with the cutoff distances of 1.0 nm and van der Waals were calculated using the particle mesh Ewald method (PME)” is not correct.

We corrected many typos.

4. The author should mark the protein name in each snapshot figure.

Since the system has only two components, it is easy to distinguish them by using different colors: green is the enzyme, red/yellow is the SpyCatcher/SpyTag. See the Figure 1 caption.

5. From the configuration of protein-protein interactions, the author should calculate the distance change between protein and protein and between residue and residue as the function of simulation time to statistically estimate the dynamics change of the proteins and residues.

For wild type, the centroid distance between the enzyme and SpyCatch/SpyTag was shortened from 4.8 to 3.2 nm during the simulations, and the distance between key residues at the interface was also shortened. For example, the distance between Lys122 and Lys347 was reduced from 1.5 to 0.7 nm, the distance between Asp124 and Lys347 was reduced from 1.4 to 0.9 nm, and that between Asp30 and Asn288 reduced from 2.6 to 0.6 nm. This was completed within the first 20 ns of MD simulation, and then remained in equilibrium, and the protein-protein interaction remained basically unchanged. In fact, the significant reduction of these distances all occurred within the first 20 ns of the MD simulations, and in the subsequent equilibrium simulations, these distances remained essentially unchanged and the protein-protein interactions also remained literally unchanged.

6. The author should do the structure alignment to compare the similar and different interactions between wild type and the mutants.

In this study, our focus is on the evolution of PPI between enzyme and SpyCatcher/SpyTag. Through the analysis of the Ring program, we can directly obtain the similarities and differences of the interactions between wild type and the mutant, without the need for structural alignment. Table S2 showed that there were van der Waals forces, hydrogen bonds, electrostatic interactions and pi-pi stacks in wild type and those in the mutant.

7. The author should analyze hydrogen bonding (including paired residues and occupancy of the hydrogen bonding) and interaction energies (values) between paired residues of the two protein to find key residues for the protein-protein interactions.

We used the Ring program (see page 8) to analyze all possible interaction (listed in Table S1) between paired residue at the interface and screened out the key residues that contribute a lot to the protein-protein interactions. In fact, Table S1 compared the PPI differences between the SpyRing complex and the random docking complex.

Reviewer #2: The SpyTag/SpyCatcher system is a useful technique to generate thermo-resistant enzymes, in which the N- and C- terminus of target enzymes were fused to the SpyTag and SpyCatcher peptides respectively. Here, Gao et al used MD simulation to study the dynamics of cyclized lichenase mediated by SpyTag/SpyCatcher and found a unique interface between lichenase and SpyTag/SpyCatcher. This result provides important insights on how the SpyTag/SpyCatcher system increases thermal stability of its target enzyme. I found this study is very interesting. However, I have two major concerns.

1. The authors state that this study provides new insights into the rational design of the SpyTag/SpyCatcher system. However, the target enzyme varies greatly in their structures and sequences. I don’t think the interface between SpyTag/SpyCatcher and lichenase here will be conserved in any other systems. Could the authors demonstrate why this feature will be a conserved property for SpyTag/SpyCatcher? Using Firefly luciferase as an example, will the SpyTag/SpyCatcher residues interacting with luciferase be the same as those interacting with lichenase?

We agree that the structures and sequences of different target enzymes are very different, so there may not be a conservative PPI between SpyTag/SpyCatcher and different proteins. Instead, this study aims to demonstrate the importance of examining the interface interaction between SpyTag/SpyCatcher and the target enzyme when considering the design of the SpyRing cyclization reaction. We have revised the statement to avoid misleading.

Compared this study with our previous study using the SnoopRing reaction to cyclize firefly luciferase, we found that in both cases, a stable interface was formed between Tag/Catcher and the target enzyme. In particular, in both cases, it had been found that electrostatic interactions involving charged residues near the termini of the Tag/Catcher play a key role in regulating the docking process and the final interface formation. These residues include Arg17 in SnoopCatcher, Lys22 in SnoopTag, Asp 30 near the N-terminus of SpyCatcher, Lys122 and Asp124 near the C-terminus of SpyCatcher (see page 10).

2. Manuscript should be written in standard English before it could be published. However, I found grammatical errors throughout the manuscript. This manuscript should be re-written in standard English before it can be published in PLOS ONE. Two of obvious errors, but not all, are listed below.

Many efforts were made to improve the manuscript, including seeking the help of native English scholars to improve it.

On page 3, The basic mechanism is that when the Tag/Catcher genes (should be peptides, genes cannot be fused to protein) are fused to the N- and C- termini (terminus) of the enzyme, they spontaneously and rapidly react to form an irreversible iso-peptide bond between ASP (Asp117) of SpyTag and LYS (Lys31) of SpyCatcher, leading to the covalent cyclization of the enzyme.

Revised.

On page 4, The cyclized enzyme has the optimum (optimal) temperature of 35℃, which was (a) 10℃ (increase) compared with the linear FLuc.

Revised.
